# The Seton

**Published:** 1902-05-03

**Authors:** 


### THE SETON.

Talking of old remedies, there have lately been several letters from medical men in praise of the seton in the treatment of migraine. One from Capt. T. E. Watson,[1] I.M.S., describes what seems to have been a complete cure of a very obstinate case, while one from Dr. Sydney Cornish,[2] of Dorking, gives somewhat fuller details regarding a case which was not only obstinate, but of such severity as to threaten the patient's reason. Moreover, it was a case in which a considerable variety of well-directed treatment carefully carried out had failed to give any marked benefit. Yet from the day on which he had a seton introduced, until the letter was written four months afterwards, the patient had had but one slight headache (on Christmas night after a particularly festive meal), and from being gloomy and suicidal he had become bright and active, and had been back at his work a month. This mode of treating migraine was brought afresh before the profession a little over a year ago by Mr. Walter Whitehead,[3] of Manchester. He said that during the last five-and-twenty years he had never failed to treat successfully the most inveterate and severe cases of migraine by the introduction of an ordinary tape seton through the skin at the back of the neck. As to the manner in which this is used, he says that the skin at the back of the neck is grasped between the finger and thumb of the left hand, and behind the fingers a long-bladed scalpel is forced so as to transfix the skin. Before the knife is removed a long probe, provided with a suitable eye, is passed through the wound using the knife as a guide. The scalpel being now withdrawn, a piece of ordinary household tape, half an inch wide, is attached by a ligature to the eye of the probe, by which it is then pulled through the wound. Four inches of tape are left free at each side, and these are gently tied together to prevent the tape being accidentally withdrawn. The tape is to be moved from side to side each day. The depth at which the seton lies varies with the thickness of the skin, and the distance between the points of entry and exit may range from one to two inches. The seton is to be worn uninterruptedly for three months at least, and should the symptoms recur a second seton ought to be introduced.

[1] Brit. Med Jour., April 19, 1902. [2] April 26, 1902. [3] Feb. 9, 1901.

